# Migrant Health Country Profile tool (MHCP-t) for transforming health data collection and surveillance in the Middle East and North African (MENA) region: tool development protocol with embedded process evaluation

**DOI:** 10.1136/bmjopen-2024-085455

**Published:** 2025-01-21

**Authors:** Stella Evangelidou, Farah Seedat, Anna Deal, Anissa Ouahchi, Taha Maatoug, Eman Elafef, Hassan Edries, Oumnia Bouaddi, Moudrick Abdellatifi, Sara Arias, Abdedayem Khelifi, Hassan Chrifi, Mohamed Douagi, Adel Abdelkhalek, Ali Mtiraoui, Wejdene Mansour, Mohamed Khalis, Mahmoud Hilali, Ibrahim Ahmed Bani, Kenza Hassouni, Bouchra Assarag, Kolitha Wickramage, Dominik Zenner, Sally Hargreaves, Ana Requena-Mendez

**Affiliations:** 1 Migrant Health, Barcelona Institute for Global Health, Barcelona, Spain; 2 The Migrant Health Research Group, St George's University of London Institute for Infection and Immunity, London, UK; 3 University of Sousse, Sousse, Tunisia; 4 University of Gezira, Gezira, Sudan; 5 Mohammed VI University of Health Sciences, Casablanca, Morocco; 6 Office Nationale de la Famille et de la Population, Tunis, Tunisia; 7 Ecole Nationale de Santé Publique, Rabat, Morocco; 8 Faculty of Veterinary Medicine, Badr University in Cairo, Badr, Egypt; 9 Higher Institute of Nursing Professions and Technical Health, Rabat, Morocco; 10 Blue Nile National Institute for Communicable Diseases, Gezira, Sudan; 11 College of Medicine, Ajman University, Ajman, UAE; 12 Laboratory of Public Health and Management, Mohammed VI Center for Research and Innovation, Casablanca, Morocco; 13 International Organization for Migration, Geneva, Switzerland; 14 Queen Mary University of London, London, UK; 15 Medicine Solna, Karolinska Institute, Stockholm, Sweden

**Keywords:** PUBLIC HEALTH, QUALITATIVE RESEARCH, Surveys and Questionnaires

## Abstract

**Abstract:**

**Introduction:**

The Middle East and North Africa (MENA) region is characterised by major health disparities and complex migration flows. Yet, because of a lack of epidemiological data, there is an urgent need to strengthen routine data collection around migrant health and to define key indicators towards migrant health monitoring. To address this problem, we aim to design and pilot test the Migrant Health Country Profile tool (MHCP-t) which can collate country-level data collection around migration health data, policies and healthcare provision.

**Methods and analysis:**

The MHCP-t development is a stepwise process that will integrate a process evaluation model with active involvement and engagement of multilevel stakeholders. First, towards the generation of indicators, qualitative field activities will be conducted in different regions in Morocco, Tunisia and Egypt with migrants (n=50 per region), migrant community leaders (n=20 per region) and professionals working with them (n=20 per region). Deductive–inductive thematic analysis will be applied to the data collected. Results from the national qualitative studies and a series of systematic reviews in the MENA region will conclude with a first draft of tool indicators which will be reviewed by national and international experts using the Nominal Group Technique. The revised indicators will be entered into an electronic data capture system and the tool will be pilot-tested by applying a mixed-methods process evaluation to examine its relevance, comprehensiveness, comprehensibility and other practical issues, such as completion time and ease of responding. Mechanisms of change will be assessed on how the participative interactions towards the tool development can trigger change at national and regional levels.

**Ethics and dissemination:**

The study protocol has been approved by the institutional review boards at the Hospital Clinic in Barcelona, Spain, the University of Sousse in Sousse, Tunisia, the University Hospital of Tanger, Morocco and Badr University of Cairo in Egypt. Findings will be disseminated in peer-reviewed journals and communications to national and regional congresses.

**STRENGTHS AND LIMITATIONS OF THIS STUDY**

- A diverse range of stakeholders, including migrants, will be engaged in the study corroborating that the tool is grounded in real-world experiences and perspectives.
- A structured, stepwise development approach will be applied including qualitative fieldwork, systematic reviews and expert feedback.
- The mixed-methods process evaluation will highlight the contextual factors, mechanisms of change and unanticipated consequences in each country.
- The pilot testing phase might not be long enough to fully evaluate the tool’s long-term impact on migrant health monitoring at the national and regional levels.
- While the development and pilot testing of the tool will take place in three countries of North Africa, the exclusion of other countries in the Middle East may limit the tool’s broader applicability across the region.

## Introduction

 The Middle East and North African (MENA) region is marked by complex mixed-migration flows with numerous countries in the region simultaneously serving as points of origin, transit and destination for multiple migrant groups.^1^ Protracted conflicts, environmental drivers and climate change, political instability and socioeconomic hardships are among the multitude of factors that influence forced displacements in the region.^2^ On the other hand, the oil-producing Gulf Countries have been challenged by the significant influence of labour migrants on their population size, with expatriates constituting between 60% and 90% of their workforce.^3^

Migrant populations in the MENA region is a heterogeneous group as reflected under the International Organization for Migration (IOM) definition: ‘an umbrella term, not defined under international law, reflecting the common lay understanding of a person who moves away from his or her place of usual residence, whether within a country or across an international border, temporarily or permanently, and for a variety of reasons’.^4^ Migrants are extremely diverse in terms of provenience, reasons to move away from usual residence and health needs as well as in terms of their rights and entitlements, with undocumented migrants facing more difficulties in accessing public healthcare facilities compared with documented migrants.^5^

The lack of epidemiological data on the major health problems affecting migrant populations, makes it difficult to promote health policies in the countries that have been hosting migrants and refugees in the region.^6^ Robust data collection around migrant health faces a number of challenges, including the fact that migrants are often excluded from health systems and health data in the host country.^7^ The MENA region is characterised by major health disparities.^8 9^ Access to life-saving assistance for displaced populations and stranded migrants in humanitarian settings continues to be of serious concern. Thus, ensuring health equity for migrants will require equal access to health services for national citizens, responsiveness of services to migrants’ needs and intersectoral attention to the social determinants of health.^10^

In order to study progress towards health equity over time, data standardisation and quantification are essential.^11^ Migrant health policymaking and programming must be driven by evidence, relying on strong data collection and surveillance at national and regional levels. A major conclusion drawn from the University College London (UCL)-Lancet Commission on Migration and Health was the urgent need to define key indicators that enable evidence-based migrant health monitoring while simultaneously strengthening global routine data collection around migrant health.^12^ Many MENA countries have not integrated migration-related indicators (eg, country of birth, legal status) into their health information systems which has resulted in a lack of comprehensive and disaggregated epidemiological data on the prevalence, outbreaks of multiple diseases and vaccination coverage. This lack of data perpetuates the challenges to map health disparities and inform evidence-based policy and service delivery in the region.^13^

### The Migrant Health Country Profile tool

The MENA Migrant Health Working Group is developing the Migrant Health Country Profile tool (MHCP-t), a tool which will enable to collate country-level data around three key domains: (1) migration health data derived from routine health information systems and other sources (eg, disease surveillance data, laboratory records); (2) policies and legal frameworks pertaining to migrant health and commitments enshrined in regional and global governance frameworks relevant to migrant health; and (3) healthcare service provision, including data on inclusion of migrants in health and vaccination services and the range of health services and initiatives that are specifically targeted towards migrants.

The tool will specifically have a transversal focus on multiple diseases (tuberculosis (TB), HIV, hepatitis B/C (Hep B/C), neglected tropical diseases (NTD), malaria, maternal and neonatal health (MHNH), non-communicable diseases (NCD), mental health), vaccine-preventable diseases and vaccination coverage, with the view to enable ministries of health to respond to the health needs of diverse mobile populations as they arise in real-time by identifying gaps in health data collection, migration-related policies and healthcare provision.

Importantly, MHCP-t will not collect disaggregated information or individual information on migrant population groups. Rather, it will gather information on how migrant health data are collected (which variables are included in the different systems), which are the existing health policies that specifically address migrant health needs and how healthcare is provided for migrant populations.

In practical terms, MHCP-t will operate as an iterative online survey with multiple questions that collect information on migrant indicators related to the three domains, health data, policies specifically related to migrant populations and access to healthcare provision (example of a question related to the health data domain would be: (1) Do you have any type of migrant indicator in your data set? (2) If yes, how are migration variables captured? The respondents of the survey will be different types of stakeholders (eg, non-governmental organisations, public health authorities, disease-specific programmes) at the country level. The survey will be eventually built on a secure web platform, the Research Electronic Data Capture and the survey will be run periodically by selected national stakeholders to monitor progress across migration-related health indicators.

### Aim and objectives

The aim of this study is to design and pilot test the MHCP-t through a process evaluation that will be carried out in Morocco, Tunisia and Egypt. We will, thus, intend to explore the differences in data collection systems, health policies and access to healthcare by different types of migrants, according to the above-mentioned IOM definition, per country. The specific objectives include:

1. To describe the methodology for the identification and assessment of the MHCP-t indicators.
2. To describe the process evaluation model integrated into the MHCP-t development phase.
3. To present the participative framework for the inclusion of multilevel and multisectoral stakeholders in the project cycle, including migrant communities through community engagement and involvement.

## Methods

### Study design

**Figure 1:**
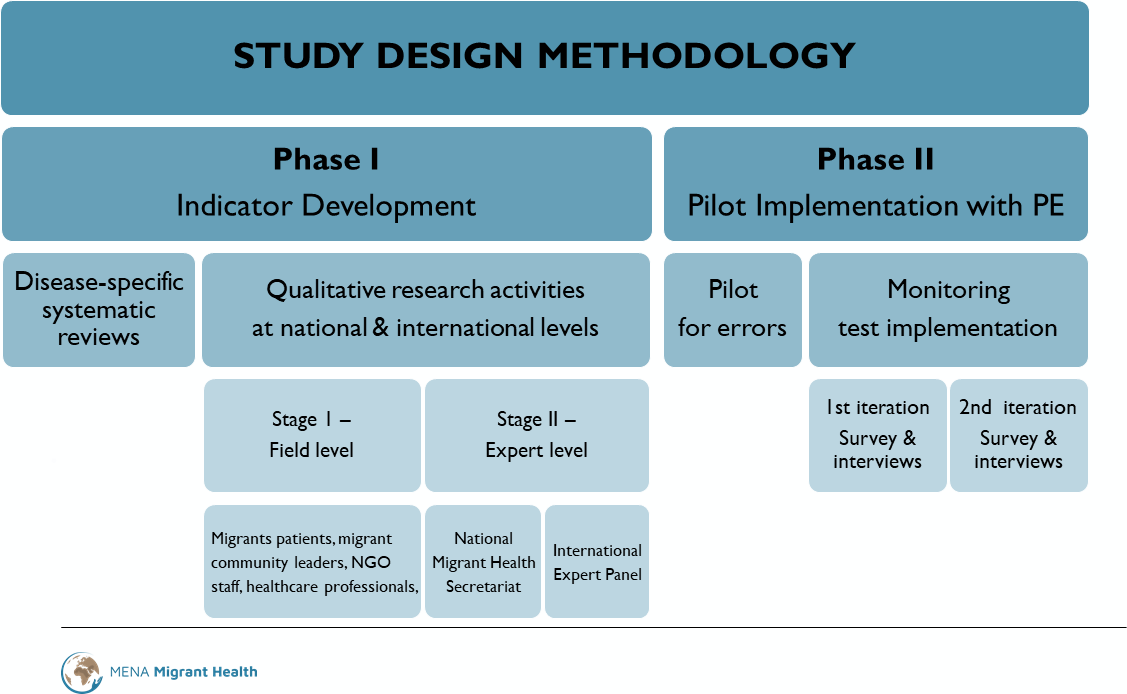
Study protocol design—methodology diagram. NGO, Non-governmental Organisation; PE, Process Evaluation

The MHCP-t development will englobe two phases: (1) indicator development and (2) pilot implementation of the test version of the tool, prior to its validation and roll-out. The estimated time period is January 2023 to March 2025. Each phase will include distinct methodological stages as illustrated in figure 1.

The embedded process evaluation will involve mixed methods towards the qualitative and quantitative understanding of the complexity of systems and the mechanisms by which interventions, such as the application of MHCP-t, may lead to impact at policy and programming levels. The sustainability of the MHCP-t innovative digital tool can only be ensured if ministries of health, NGOs, civil society and migrant communities are engaged and involved since the initial steps of its conceptualisation and development.

**Figure 2:**
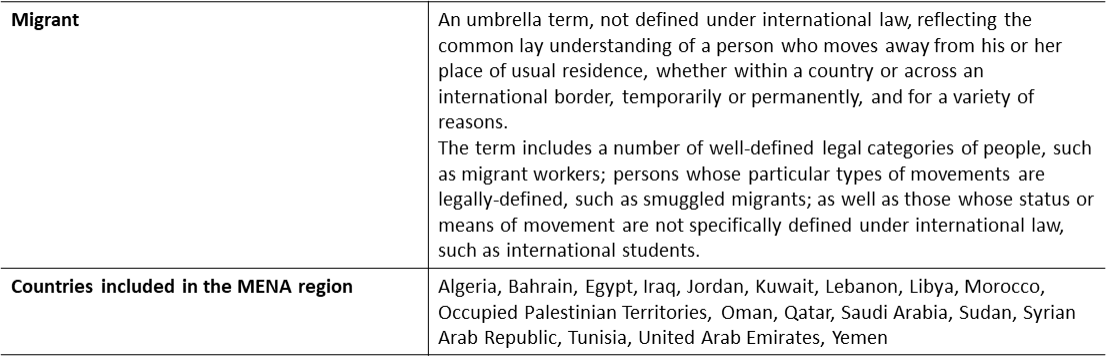
Definitions adopted from the International Organization for Migration.^1 4^ MENA, Middle East and North Africa

Definitions for migrant and the MENA region are described in figure 2.^1 4^

### Phase I: indicator development

Indicator development for the tool will be a twofold process that includes: (1) a suite of disease-specific systematic reviews in the MENA region with the purpose of synthesising the burden and clinical outcomes of specific diseases relevant to the project on migrant populations, the protocol of which has been published elsewhere;^14^ and (2) a series of qualitative research activities at field level, while also engaging national and international experts on migrant health. The qualitative methodology for the indicators’ development will be also carried out in two stages.

First, the views and perspectives of migrants, healthcare professionals and NGO staff will be sought around health, healthcare and vaccination service provision for migrants. The qualitative study findings will be triangulated with the systematic review findings towards the generation of the first draft of indicators. Second, the draft of the tool will be presented to national and international experts on migrant health, migration policy and evaluation tools, for review and refinement. Lastly, a consensus will be established at the national-level on the final version of the tool indicators for pilot testing.

#### Stage 1: at field level

##### Study settings

For the qualitative methodology of the tool development, Morocco, Tunisia and Egypt were chosen due to pre-existing network capacities and political stability, allowing the prompt planning and implementation of field activities. The research teams in Morocco, Tunisia and Egypt decided on the study sites for data collection. In Morocco, qualitative study activities will take place in Rabat, Casablanca, Tanger, Oujda, Agadir and Dakhla. In Tunisia, the activities will take place in Tunis, Medenine, Sousse and Sfax. In Egypt, the proposed sites are in Cairo, Alexandria and Aswan. These selected locations have a high density of migrants.

##### Participants and recruitment

At each selected study location, migrant community leaders, NGO staff, health professionals in the public healthcare system and migrants themselves will be engaged in the qualitative research part of the project.

10–20 individual interviews with migrant community leaders and two to four focus group discussions (FGD) with NGO staff or health professionals at the national healthcare system who work with migrants will be done. In case FGD cannot be organised because of participants’ limited availability, approximately 10 individual interviews will take place with them. Migrant community leaders and professionals working with migrants can provide their assessments and insights on the main health and social needs of migrants and to what extent NGO and national healthcare systems are ready to respond to those needs.

Migrant men and women will be invited to express their needs and views on the healthcare system separately in two to four FGD at each site. The criterion for the group composition will be sex, legal status and language (French, English or Arabic).

The maximum number of participants at each FGD activity will be 8–10.

Additionally, 5–10 in-depth individual interviews (IDI) will be conducted with migrant patients for every health condition that is of research interest in the project teams of Morocco, Tunisia and Egypt (NCD/mental health, vaccination, TB, MHNH, HIV, Hep B/C, malaria and NTD).

The number of activities stated above only serves as a guide, since data saturation at each site will determine the final numbers. Topic guides for each type of session will be developed depending on the profile of interviewees and will be piloted before their application. One topic guide will be developed for the FGD with migrants, another one for its use with migrant community leaders and a third one for NGO staff and healthcare professionals. The objective of all three topic guides will be to explore migrants’ health needs, help-seeking behaviours, barriers to healthcare access and suggestions for improvement from three perspectives, that is, migrants’, community leaders and professionals’ views working with them (online supplemental annex 1). Disease-specific topic guides will be developed for in-depth interviews with migrant patients. In-depth interviews with migrant patients with TB, HIV, patients with Hep B/C, patients with malaria and NTDs, migrant patients with NCDs and mental health problems as well as pregnant/postpartum migrant women and migrants who have been vaccinated or refused to be vaccinated in the country will be interviewed on their experiences regarding their health condition and the healthcare assistance received in the country.

The identification and recruitment of migrant community leaders, NGO staff and migrants will be done through collaboration with national and international NGOs in all countries. Migrant patients for each health condition will be recruited by the same NGO and/or through the regional health authorities. Health professionals at public healthcare system facilities will be reached through regional health authorities too.

##### Procedure

The qualitative research activities will be conducted by six predoctoral researchers, two in every partner country in North Africa (Morocco, Tunisia, Egypt) with the supervision of national and international migration health experts. At each qualitative activity, one predoctoral student will moderate the session in any of the three project languages (Arabic, French, English) and the other one will take field notes, based on a predetermined grid, on non-verbal communication, group dynamics and a summary of key ideas expressed. Session duration will be approximately 40 min for individual interviews and IDI and 2 hours for FGD. The sociodemographic characteristics of participants will be collected. Specifically, for migrants: country of origin, age, sex, time of stay in the country, level of education, occupation, legal status, while for NGO staff and healthcare professionals: country of origin, age, sex, job position in the organisation, years of experience working with migrants as well as name, type and location of institution/organisation. IDI and FGD will be audio-recorded after written informed consent is obtained from participants. Audio recordings of the sessions will be destroyed once verbatim transcriptions are completed. All transcripts will be anonymised (for participants and their institutions, if applicable) before analysis.

##### Data analysis plan and theoretical approach

The method of analysis chosen for this study is a hybrid approach, introduced by Federay and Muir-Cochrane,^15^ of qualitative methods for thematic analysis. It will incorporate both the data-driven inductive approach of Boyatzis^16^ and the deductive a priori template of codes approach outlined by Crabtree and Miller.^17^ This hybrid approach complements the study objectives by allowing the tenets of social phenomenology to be integral to the process of deductive thematic analysis while allowing for themes to emerge directly from the data using inductive coding.

The predoctoral researchers will familiarise themselves with the transcriptions in the language the activities took place. Text will be then segmented into meaningful codes into English that could be categorised and grouped into conceptual patterns or themes (inductive approach). Any cultural–linguistic issues in this first step of coding will be addressed at cross-country level. The a priori framework used for the deductive approach will be Levesque’s conceptual framework of patient-centred healthcare access^18^ which views access as the opportunity to identify healthcare needs, to seek healthcare services, to reach, to obtain or use healthcare services and to actually have a need for services fulfilled. In this framework, there are five dimensions of accessibility: (1) approachability, (2) acceptability, (3) availability and accommodation, (4) affordability, (5) appropriateness, which correspond to five corollary groups of abilities: (a) ability to perceive, (b) ability to seek, (c) ability to reach, (d) ability to pay, (e) ability to engage.

The qualitative data analysis will be conducted using NVivo software. Cross-country comparative analysis will be carried out by sharing the generated codebooks across the study sites.

### Stage 2: at experts’ level

Two project researchers will summarise the qualitative study results with the systematic review findings^19^ and set the first draft of indicators. The MENA Migrant Health Working Group has predetermined three domains for the MHCP-t: Migrant health data, health policies and healthcare provision under which indicators will be grouped. The analytical praxis used by the researchers will translate systematic review findings into indicators related to migrant health data and health policies, while qualitative research findings will inform indicators concerning the domain of healthcare provision. National and international experts will review the drafted items per domain.

#### Participants

At the national level, the first draft of indicators will be presented and reviewed by the Migrant Health Secretariat (MHS) in Morocco, Tunisia and Egypt. This refers to a group of key stakeholders (national migration health experts, NGO and civil society representatives, migrant community representatives) who will be responsible for the revision of the tool indicators and their implementation at the country level. The MHS will be affiliated with the national Ministries of Health, since they will be the ultimate drivers for the tool development and implementation in the long term.

At international and regional levels, a panel of international professionals with expertise in migrant health, the multiple diseases the project focuses on and the development of indicators for public health surveillance will be contacted.

#### Procedure

The members of the MHS in Morocco, Tunisia and Egypt will be invited to review and refine the first draft of indicators. As a step to establish the content and face validity of the indicators, that is, their relevance and comprehensiveness, prior to the tool’s pilot testing and statistical validation, we will apply the Nominal Group technique (NGT)^20 21^ with national and international experts. This is a discursive approach method for collecting and processing ideas and opinions in a collaborative group. It entails a structured and controlled group decision-making process with continuous visualisation of the discussion development. It builds a non-judgemental environment of stimulating a variety of ideas, allowing multiple points of views to be expressed. It has four main stages: silent generation of ideas, round-robin recording of ideas, clarification and voting/ranking. NGT, in contrast to other interacting group methods, provides a setting in which participants work alone initially and each individual’s contributions are later pooled among other stakeholders. Unlike focus groups, NGT assures a balanced input from all participants and takes advantage of each person’s knowledge and expertise.

Each MHS member or every small group will be asked to reflect on the first draft of indicators on an interactive virtual platform. For each single indicator formulated as a question in the tool or group category of indicators, participants will need to rate its fit under the domain it has been assigned to on the platform. The rating scale will range from 1 (strongly disagree) to 5 (strongly agree). Room for proposal of new indicators will be made. Any new suggested indicators, together with those which have created strong disagreements based on stages 1 and 2 of the study procedure, will be put at the plenary for final voting towards a group consensus within the MHS which includes the engagement of representatives of migrant communities.

On the basis of the rating and voting system from the MHS in Morocco, Tunisia and Egypt some indicators may be deleted and some others may be added to the MHCP-t. A second draft of the indicators will be then presented to the panel of international and regional experts and will be assessed with the same methodology on an online virtual platform. Further revisions will be made and a more updated version of the tool will be returned to the MHS in each country for a final consensus.

Each of the two nominal group sessions will last 3 hours. All MHS members will provide informed consent forms for their participation. Sessions will not be audio-recorded, but all visual representations of the discussion will be saved while keeping the anonymity of participants.

#### Data analysis

The advantages of the NGT include quick outcomes to be obtained by the end of each session, the large number of ideas to be generated and ‘shared ownership’ of the tool developed by participants. The visual representations of the discussions from both MHS sessions, for example, rankings per indicator and graphs, will allow the comparison between the two in terms of the context-specific content of the indicators. Any variation of the indicators will be brought to the international panel of experts so that a consensus can be made.

### Phase II: pilot implementation with integrated process evaluation

The revised set of indicators will be used in the pilot version of the MHCP-t which will be built on an electronic data capture platform.

#### Participants

The test version of the MHCP-t will be sent to approximately fifteen respondents selected by the MHS in each country. The respondents need to be specialists in migrant health in the public sector or at NGOs at the country level and should not have been in the prior steps of the tool development. Their responses will refer to the data collection system of the institution/organisation they represent.

#### Procedure

First, the revised MHCP-t will be piloted for errors and then, we will monitor its test implementation with a mixed-method process evaluation methodology.

During a period of 1 month, the tool respondents will be asked to fill in the tool on the electronic data capture platform and complete a short survey on (1) the relevance of the indicators to the field of migrant health and the country context, (2) the comprehensiveness of the tool, whether there are any missing items while providing room for suggestions, (3) the comprehensibility of instructions, indicators/items and response options and (4) practical issues, such as completion time, ease of responding. The responses will be rated on a 5-point Likert scale. Additionally, a third of the respondents will be asked to participate in semistructured interviews where they will be asked about the positive aspects of the MHCP-t, the aspects to be improved, how the tool can improve their area of work in migrant health and the extent to which it can become an integral part of their periodic reporting. Survey respondents and interviewees will be asked for a series of sociodemographic variables (age, sex, occupation, type of organisation/institution, position, years of work in the organisation/institution, years of work in the migration health sector).

During the following 2 months, the tool will be further updated based on the survey and interview results. A second iteration of the tool will take place with the same respondents during a period of 1 month. The same short survey will be administered in order to track any changes made. Another third of respondents will be interviewed at the end of the second iteration. The final version of the tool will be ready for its statistical validation (validation strategy to be published separately).

#### The embedded process evaluation model

We consider MHCP-t a public health intervention according to the international classification of health interventions.^22^ Process evaluation methodology allows the logical modelling of intervention processes and outcomes. The UK Medical Research Council guidance for the development and evaluation of complex interventions highlights the importance of programme theory at the micro-project level (theory of change, at the macro-policy level) and the role of context in shaping the intervention development.^23^ Programme theory encompasses any set of causal assumptions surrounding how defined actions produce intended or unintended consequences. The ecological fit of MHCP-t as a public health intervention with the systems, whose functioning they attempt to change, is also a significant component of the process evaluation. All deliberate system changes are founded on a theory of change.^24^ Contextualised and system-led theories of change will add value to our process evaluation design.^25^

**Figure 3:**
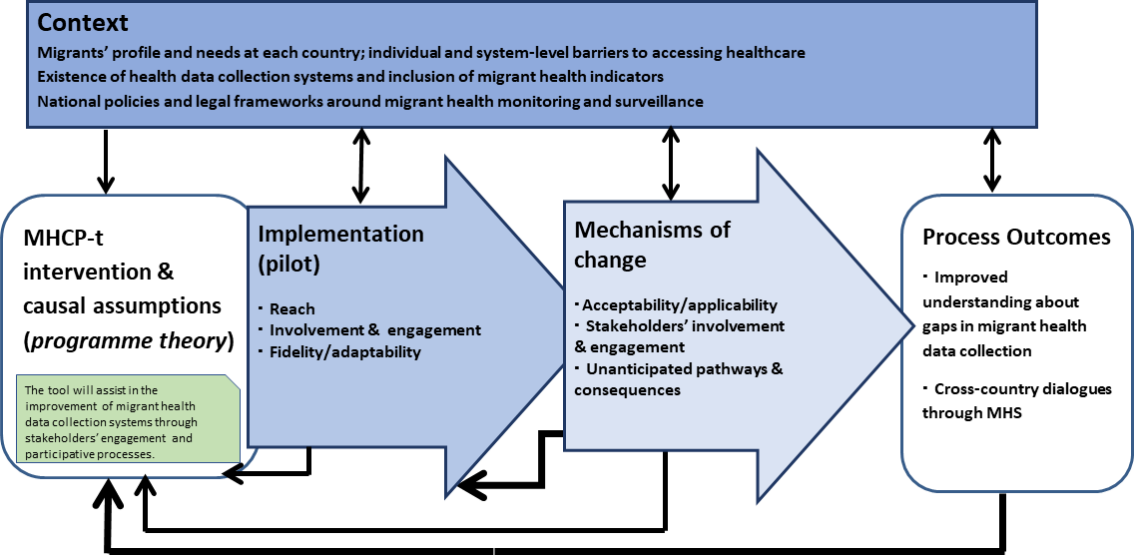
Process evaluation framework for the MHCP-t pilot implementation. MHCP-t, Migrant Health Country Profile tool; MHS, Migrant Health Secretariat.

Figure 3 depicts the logic model of the process evaluation components: context, implementation characteristics, mechanisms of change/impact and process outcomes. The embedded process evaluation will broaden the scope of the tool development by including its impact identification, theorising how it works, taking into account the contextual factors where it is implemented and how it can contribute to system change and policymaking.

The context evaluation will examine how external factors can influence the tool development, such as migrant health needs and access barriers to healthcare, migrant disease burden and existing policies around migrant health.

We will conduct a multicomponent assessment of the pilot implementation process evaluation comprising: (1) reach of the tool to respondents, invited by the MHS focal point, (2) involvement and engagement of migrants in the review of the drafted indicators, of MHS members and of international experts, (3) fidelity/adaptability of the MHCP-t core structure based on country-specific recommendations and proposed editions/additions.

Mechanisms of change/impact focus on how the tool and participative interactions towards its development can trigger change, by assessing (1) the tool’s acceptability/applicability by respondents, (2) stakeholders’ involvement in the creation and establishment of the MHS at country-level and migrants’ involvement in those, (3) unanticipated pathways and consequences based on MHS potential new initiatives among the multidisciplinary stakeholders.

Table 1 enlists the previewed measures and data sources for each process evaluation component under each process component.

**Table 1:** Process evaluation dimensions, constructs, variables along data sources

Process dimensions	Process construct	Process variable	Data source
Context	Fit of the intervention at national level	Migrant health needs and access barriers to healthcare	Country-specific and disease-specific qualitative study findings
	Fit of the intervention at regional level	Migrant disease-specific burden	Disease-specific systematic reviews at MENA region
Implementation (pilot)	Reach	No of tool respondents reached/no of respondents contacted	Documentary analysis by MHS focal point
		No of surveys completedNo of individual interviews conducted	Field note records
	Involvement and engagement	No of sessions conducted with migrants’ representatives for the review of indicators	Steering committee notes
		No of MHS members assisting in the NGT sessions/no of MHS members overall	Documentary analysis by MHS focal point
		No of international experts contacted/no of international experts invited	Documentary analysis
	Fidelity/adaptability	Extent of adherence to MHCP-t core structure versus extent of adaptability according to country specificities	Field notes of NGT
		No of changes proposed after first & second tool iterations while assessing comprehensiveness and comprehensibility of the tool	Surveys and interviews with respondents
Mechanisms of change	Acceptability/applicability	Extent of acceptability of the tool	Surveys and interviews with respondents
		Extent of applicability of the tool in the field of migrant health	Surveys and interviews with respondents
	Stakeholders’ involvement and engagement	Creation and establishment of MHS	Documentary analysis
		Migrants’ involvement at MHS	Individual interview with migrant community leaders
	Unanticipated pathways and consequences	MHS new initiatives among multidisciplinary stakeholders	Documentary analysis

MENAMiddle East and North AfricaMHCP-tMigrant Health Country Profile toolMHSMigrant Health SecretariatNGONon-Governmental OrganisationNGTNominal Group techniquePEProcess Evaluation

Once the tool is validated in at least one of the participant countries, it will be then rolled out to other countries in the MENA region following a rigorous validation process which will assess the tool’s content validity and contextual fit. However, this is beyond the scope of the present study protocol and it will be developed separately on upcoming publications of our Consortium.

#### Process evaluation data analysis

Data collection tools will consist primarily of documentary analysis by MHS focal points, field notes of the nominal groups with MHS members and international experts as well as surveys and individual interviews with the respondents during the pilot implementation.

The quantitative data, generated by the tool administration and the surveys during its pilot test will be analysed on the statistical software package Stata. We will explore the distribution of responses overall and by different subgroups within and across counties as well as the results of the satisfaction surveys completed by the respondents. Statistical differences in the respondents’ tool ratings on the satisfaction survey will be explored by job profile, sex and country using ordinal logistic regression. To preserve anonymity, a study code will be given to each respondent. Quantitative data will be entered into a database containing fields for each process dimension.

The MENA MH Consortium had decided not to include any composite scoring to the MHCP-t, as it is not acceptable to stakeholders the categorisation of countries or institutions within a country which may lead to unnecessary comparisons. The MHCP-t is to be used as a mapping and reflective exercise on health data information systems, migration-related policies in place and main aspects of migration-related healthcare provision to strengthen systems within countries and not to ‘name and shame’.

For the qualitative data generated by the individual interviews, the NVivo software will be used to support data management and analysis. Thematic analysis will be conducted. Two doctoral students will code and categorise the transcripts to construct one coding framework. A priori codes that map onto the process evaluation components will be included in the coding framework, along with novel codes that emerge from the data. Once all transcripts are coded, candidate themes will be identified for each data set and overall. Anonymised data will be presented in the form of quotes to illustrate each theme.

### Patient and public involvement

The project will maintain solid partnerships with academia, ministries of health, NGOs, civil society and—importantly—migrant communities who are the main beneficiaries of this research. We are committed to embedding participatory action research and community engagement and involvement in all stages of this project. The creation of the MHS in each country will enable country ownership and sustainability of the tool and further, it will support its roll-out both within and beyond the MENA region. MHS will include two migrant community leaders. All project phases will be supported by the advice of migrant representatives in the periodic steering committees.

The involvement and engagement of migrants and stakeholders from various structures and countries will create a positive impact on the participative development and sustainable implementation of the tool.^26^

## Ethics and dissemination

### Ethical considerations

The study protocol has been approved by the ethics committees of the Hospital Clinic, Barcelona, Spain (HCB/2022/0655), the University Hospital of Tanger, Morocco (09/2022), the University of Sousse, Medicine Faculty, Tunisia (CEFMS 157/2023) and Badr University of Cairo (BUC-IACUC-231217-52/53), Egypt.

Informed consent will be obtained through discussion between members of the research team and potential participants and supported by written participant information sheets (PIS). PIS will detail the nature of the research, our study objectives. The right to decline to participate or to withdraw consent at any stage of the research will be explicitly stated. Consent forms and PIS will be available in Arabic, English and French languages. The opportunity will be given for participants to ask any questions about the scope of the research or their rights as participants during the consent process. The anonymity of responses and confidentiality of the data will be also highlighted.

### Dissemination

The promoter and investigators commit to publish the results of the study in journal articles, other scientific publications and communications to congresses. We will design tailored communications to migrant communities and other key stakeholders nationally and regionally regarding our research findings.

### Strengths and limitations

A strength of our study is the engagement of a diverse range of stakeholders, including migrants, ensuring that the tool is grounded in real-world experiences and perspectives, making it comprehensive and relevant.

We do recognise that the migrant definition we use is very broad and it may include migrant groups that are not of primary interest. However, our tool is not intended to address the health burden of vulnerable migrants but rather to map how the indicators needed to do this assessment are being collected throughout each country. Therefore, we used the broad IOM migrant definition, since ‘being a migrant’ may be easier to capture in health information systems and/or health surveillance systems. In other words, it is not easy to differentiate expatriates versus other groups of migrants unless the country of birth or last country of residence is collected, since health information systems usually do not reflect other vulnerability indicators per se (eg, poverty).

Our tool precisely aims to explore any type of migrant indicators and further, how such indicators are collected and whether they are able to capture vulnerabilities among different migration groups. For example, the indicator ‘country of birth’ would be able to differentiate people from high versus low-income countries and ‘migrant status’ would assess the information related to the legal status of the migrants. The purpose of the tool is that in the long-term migrant indicators of surveillance systems and data sets can be redefined by selecting those appropriate to capture adequately the migrant vulnerabilities across different migrant groups.

The tool development will follow a structured, stepwise approach that includes qualitative fieldwork, systematic reviews and expert feedback which can enhance the robustness and scientific rigour of the tool design. The mixed-methods process evaluation will highlight the contextual factors which can influence the development of the tool and will underline the triggered mechanisms of change by assessing the tool’s acceptability, applicability and unanticipated consequences in each country. While the study plans to assess mechanisms of change triggered by the participative interactions, the pilot testing phase might not be long enough to fully evaluate the tool’s long-term impact on migrant health monitoring at the national and regional levels.

The development and pilot testing of the tool will take place in three countries of North Africa, the exclusion of other countries in the Middle East may limit the tool’s broader applicability across the region, as the migration and health contexts can vary significantly from country to country. On its successful development in the study countries, the MHCP-t will be rolled out in other countries of the region and beyond, after appropriate contextual validation.

## supplementary material

10.1136/bmjopen-2024-085455 online supplemental file 1
