# Supplementary material for: Migrant Health Country Profile tool (MHCP-t) for transforming health data collection and surveillance in the Middle East and North African (MENA) region: tool development protocol with embedded process evaluation
Source: BMJ Open. 2025 Jan 21;15(1):e085455. doi: 10.1136/bmjopen-2024-085455 (PMC11784429; doi:10.1136/bmjopen-2024-085455)
Supplement: online supplemental file 1 [file bmjopen-15-1-s001.docx]

**Annex 1.** Topic Guides for migrants, migrant community leaders and NGO/healthcare staff

**Index:**

- 1. Topic Guide for Focus group discussion with migrants
  2. Topic guide for semi-structured individual interviews with migrant community leaders
  3. Topic guide for semi-structured with NGO/healthcare staff working with migrants

**Objective:** To explore migrants’ health needs, help seeking behaviors, barriers to healthcare access and suggestions for improvement from three perspectives, i.e. migrants’, migrant community leaders´ and professionals´ views working with them

- 1. **Topic Guide for Focus group discussion with migrants**

• 1. What are your main health needs/main health problems?

• 2. What type of help you usually seek for your health problems?

• 3. What type of health services do you usually visit? What institution/organization (public, private, NGO) and (b) how would you describe the quality of the healthcare provision? What may be the barriers to healthcare? How would you describe a good quality care provision?

• 4. How common may be the following health condition in your community: *NCDs(diabetes, cardiovascular problems, respiratory, cance), mental health, HIV, Hep B/C, TB, malaria, NTD (leishmaniasis, Scabies, dengue, and soil-transmitted Heliminthis), maternal and neonatal health issues*. How would you describe the healthcare access and quality of care provision for these conditions?

• 5. Have you been vaccinated since you arrived in the country? If yes, how would you describe the vaccination services? If not, were vaccinations offered to the community? What may be the reasons you refused?

•6. What are your suggestions for increasing migrant healthcare access and improving quality of care?

- 1. **Topic guide for semi-structured individual interviews with migrant community leaders**

• 1. Describe your community to us. What is your role in the community?

• 2. What are the main health needs/main health problems of the people in your community?

• 3. What type of help do they usually seek for their health problems?

• 4. What type of health services do they usually visit? What institution/organization (public, private, NGO) and (b) how would you describe the quality of the healthcare provision? What may be the barriers to healthcare? How would you describe a good quality care provision for your community group?

• 4. How common may be the following health condition in your community: *NCDs (diabetes, cardiovascular problems, respiratory, cance), mental health, HIV, Hep B/C, TB, malaria, NTD (leishmaniasis, Scabies, dengue, and soil-transmitted Heliminthis), maternal and neonatal health issues*. How would you describe the healthcare access and quality of care provision for these conditions?

• 5. To what extent people in your community get vaccinated since they arrive in the country? What are the common beliefs around vaccination (child, adult, COVID) in your community? How would you describe the vaccination services in the country?

•6. What are your suggestions for increasing migrant healthcare access and improving quality of care?

- 1. **Topic guide for semi-structured with NGO/healthcare staff working with migrants**

• 1. What is the profile of migrants that visit your services?

• 2. What are their main health needs/main motives for consultation?

• 3. What type of help do they usually seek for their health problems?

• 4. What type of health services do they usually visit? What institution/organization (public, private, NGO) and (b) how would you describe the quality of the healthcare provision for migrants? What may be the barriers to healthcare access? How would you describe a good quality care provision for migrants in the country?

• 4. How common may be the following health condition among the migrants they visit your services/facilities: *NCDs(diabetes, cardiovascular problems, respiratory, cance), mental health, HIV, Hep B/C, TB, malaria, NTD (leishmaniasis, Scabies, dengue, and soil-transmitted Heliminthis), maternal and neonatal health issues*. How would you describe the healthcare access and quality of care provision for these conditions?

• 5. To what extent migrants get vaccinated since they arrive in the country? How would you describe the vaccination services available to migrants (child, adult, COVID)in the country?

•6. What are your suggestions for increasing migrant healthcare access and improving quality of care?
