# Supplementary material for: Migrant Health Country Profile tool (MHCP-t) for transforming health data collection and surveillance in the Middle East and North African (MENA) region: tool development protocol with embedded process evaluation
Source: BMJ Open. 2025 Jan 21;15(1):e085455. doi: 10.1136/bmjopen-2024-085455 (PMC11784429; doi:10.1136/bmjopen-2024-085455)
Supplement: Supplementary file 2 [file bmjopen-2024-085455.reviewer_comments.pdf]

PEER REVIEW HISTORY

BMJ Open publishes all reviews undertaken for accepted manuscripts. Reviewers are asked to complete a checklist review form and are provided with free text boxes to elaborate on their assessment. These free text comments are reproduced below.

ARTICLE DETAILS

Title (Provisional)

Migrant Health Country Profile tool (MHCP-t) for transforming health data collection and surveillance in the Middle East and North African (MENA) region: Tool development protocol with embedded process evaluation

Authors

Evangelidou, Stella; Seedat, Farah; Deal, Anna; Ouahchi, Anissa; Maatoug, Taha; Elafef, Eman; Edries, Hassan; Bouaddi, Oumnia; Abdellatifi, Moudrick; Arias, Sara; Khelifi, Abdedayem; Chrifi, Hassan; Douagi, Mohamed; Abdelkhalek, Adel; Mtiraoui, Ali; Mansour, Wejdene; Khalis, Mohamed; Hilali, Mahmoud; BANI, IBRAHIM AHMED; HASSOUNI, Kenza; Assarag, Bouchra; Wickramage, Kolitha; Zenner, D; Hargreaves, Sally; Requena-Mendez, Ana; Migrant Health Working Group, MENA

VERSION 1 - REVIEW

**Reviewer**	**1**
**Name**	**Espinoza, Juan**
**Affiliation**	**Ann and Robert H Lurie Children's Hospital of Chicago, Stanley Manne Children's Research Institute**
**Date**	**27-May-2024**
**COI**	**None.**

- The authors define the terms migrant in Figure 2, but this occurs too late in the paper. The introduction sometimes conflates groups of individuals like economic migrants and refugees, but I'm sure that the authors know that these are two very different classes of people facing different kinds of barriers. It would be helpful to clarify right away who is and is not included in the scope of "migrant" for this project. It would also be beneficial for the authors at least discuss how different types of migrants (refugees, asylum seekers, economic migrants, immigrants) differ in terms of their health barriers, and justify why it still makes sense to look at them all together.

- The description of the MHCP-t still does not give me a clear understanding of the form or function of the tool. The authors are undertaking mixed methods to develop and refine it, but I imagine they have some framing of the tool in mind. It would be helpful to share that.

For example, is it a survey? An interactive document? Does it come with data or ask the user of the tool to populate certain elements?

- I understand that the authors are starting in Morocco, Tunisia, and Egypt because of existing relationships and infrastructure. This is reasonable, but it would be god to acknowledge that all three of these countries are in North Africa and that the migrant communities may be different and face different challenges than those in the Middle East; e.g., there are many more Nepali migrant workers in the gulf states than in North Africa, and they have unique challenges do to Nepal's laws around migrant work.

- As I was reading the description of indicators, I kept looking for a quantitative component. When I think of indicators, I think of composite metrics like the human development index, Wealth Inequality measure, World Press Freedom Index. All of these have more quantitative components, sometimes with robust statistical methods. I did not see this discussed, and I'm left wondering what if this will happen in a later phase of the project, or is simply not a part of this project at all. Quantitative measures are alluded to in the beginning but then never discussed again.

- I do not see a discussion of limitations, but that may not be required for protocol papers, in which case please ignore this comment.

**Reviewer**	**2**
**Name**	**Koppenol-Gonzalez, Gabriela**
**Affiliation**	**War Child Alliance , Research & Development**
**Date**	**21-Aug-2024**
**COI**	**I understand and have no competing interests.**

Dear authors,

First of all, I enjoyed reviewing this manuscript very much, I think it describes very important work addressing a serious gap in global mental health and the participatory approach is very much needed. Overall, the manuscript is well written and the protocol is clear. However, while reading the manuscript some questions came up and addressing them in the text may clarify some aspects to the reader.

I suggest to expand the Introduction with a bit more explanation about the tool. The problem is clear, but how the tool will address that problem can use some additional information. For example, it is stated on pp.4-5 that the tool will focus on multiple diseases and "enable ministries of health to respond to the health needs of diverse mobile populations as they arise in real-time". How is that envisioned? Will the tool enable a link between health data and populations as they change locations? If so, how? Is the tool like a database that can be consulted to obtain this information? And how will that be done without disaggregated information on migrant population groups?

On p.8 under Procedure it says that the name of the NGO staff and healthcare professionals will be collected. Will the reports be anonymous by linking the names to an ID in a separate file? If so, please mention this in the text.

On p.9 under Stage 2: At experts' level it says that the qualitative study results and the systematic review findings will be summarized and compared, but what will be compared? It seems as if the qualitative results will be used for two of the domains, while the review findings will be used for the third domain. I suggest to clarify here what the comparison entails.

Minor: on p.9 line 34 it says Tunisia twice, should be Egypt?

On p.10 second paragraph it is explained how the indicators from field level will be used as input for the expert level rating, that there is room for proposal of new indicators and that strong disagreements will be put at the plenary for group consensus. I was wondering what the procedure will be in case there are many strong disagreements between the indicators from stage 1 and those from stage 2? Could you please add that to this part?

On p.11 line 5, could you please add that the data capture platform will be used to collect data on the tool? It only became clear to me later in the text.

On p.13 line 17 it says "between the two countries", should that be "three countries"? Also on line 18, it is briefly mentioned that inferential statistics will be performed and reported. I suggest to specify what type of inferential statistics and with what aim.

I'm very happy to see the part about migrants' and stakeholders' involvement on p.13 and encourage you to write about that too, after conducting the study, Best of luck!

VERSION 1 - AUTHOR RESPONSE

Reviewer 1

Dr. Juan Espinoza, Ann and Robert H Lurie Children's Hospital of Chicago

Comments to the Author:

- The authors define the terms migrant in Figure 2, but this occurs too late in the paper. The introduction sometimes conflates groups of individuals like economic migrants and refugees, but I’m sure that the authors know that these are two very different classes of people facing different kinds of barriers. It would be helpful to clarify right away who is and is not included in the scope of “migrant” for this project. It would also be beneficial for the authors at least discuss how different types of migrants (refugees, asylum seekers, economic migrants, immigrants) differ in terms of their health barriers, and justify why it still makes sense to look at them all together.

Response: Thank you for this comment that really uncovers the broad perspective of the proposed tool. After the first paragraph in the "Introduction", we included a second one on the heterogeneity of the migrants globally and in the MENA region (p.4):

"Migrant populations in the MENA region is a heterogeneous group as reflected under the International Organisation for Migration (IOM) definition: "*an umbrella term, not defined under international law, reflecting the common lay understanding of a person who moves away from his or her place of usual residence, whether within a country or across an international border, temporarily or permanently, and for a variety of reasons*"(4). Migrants are extremely diverse in terms of provenience, reasons to move away from usual residence and health needs as well as in terms of their rights and entitlements, with undocumented migrants facing more difficulties in accessing public healthcare facilities compared to documented migrants (5)".

The MHCP-t, as a monitoring and surveillance tool of migration-related indicators, is inclusive to all types of migration, according to the abovementioned IOM definition.

Further, we have added the additional statement under "Aim and objectives" justifying why it makes sense to look at them altogether (p.6):

"We will, thus, intend to explore the differences in data collection systems, health policies and access to healthcare by different types of migrants, according to the abovementioned IOM definition, per country".

- The description of the MHCP-t still does not give me a clear understanding of the form or function of the tool. The authors are undertaking mixed methods to develop and refine it, but I imagine they have some framing of the tool in mind. It would be helpful to share that. For example, is it a survey? An interactive document? Does it come with data or ask the user of the tool to populate certain elements?

Response: For a more detailed description of the MHCP-t we have added a paragraph at the end of p.5:

*"*In practical terms, MHCP-t will operate as an iterative online survey with multiple questions that collects information on migrant indicators related to the three domains, health data, policies specifically related to migrant populations, and access to healthcare provision (example of a question related to the health data domain would be: (a) Do you have any type of migrant indicator in your dataset? (b) If yes, how are migration variables captured ?). The respondents of the survey will be different type of stakeholders (e.g., NGOs, public health authorities, disease specific programmes) at country level. The survey will be eventually developed on a secure web platform, the Research Electronic Data Capture (REDCap), and the survey will be run periodically by selected national stakeholders to monitor progress across migration-related health indicators".

- I understand that the authors are starting in Morocco, Tunisia, and Egypt because of existing relationships and infrastructure. This is reasonable, but it would be good to acknowledge that all three of these countries are in North Africa and that the migrant communities may be different and face different challenges than those in the Middle East; e.g., there are many more Nepali migrant workers in the gulf states than in North Africa, and they have unique challenges do to Nepal's laws around migrant work.

Response: We completely agree with your observation in this regard. Political stability has been an additional factor to pre-existing network capacities for the selection of the participant counties. We have now added both criteria under "Study settings" in Methodology (p.7).

"For the qualitative methodology of the tool development, Morocco, Tunisia and Egypt were chosen due to pre-existing network capacities and political stability, allowing the prompt planning and implementation of field activities".

Further, we do acknowledge that all three countries are located in North Africa. The exclusion of countries in the Middle East has been included as a study limitation under "Strengths and limitations of this study" (p.3) and under the new general section "Strengths and Limitations" in the text (p.15):

"While the development and pilot testing of the tool will take place in three countries of North Africa, the exclusion of other countries in the Middle East may limit the tool's broader applicability across the region, as the migration and health contexts can vary significantly from country to country."

Further, at the end of the section "The embedded process evaluation model" (p.13), we have added:

"Once the tool is validated in at least one of the participant countries, it will be then rolled-out to other countries in the MENA region following a rigorous validation process, which will assess tool's content validity and contextual fit. However, this is beyond the scope of the present study protocol and it will be developed separately on upcoming publications of our Consortium".

- As I was reading the description of indicators, I kept looking for a quantitative component. When I think of indicators, I think of composite metrics like the human development index, Wealth Inequality measure, World Press Freedom Index. All of these have more quantitative components, sometimes with robust statistical methods. I did not see this discussed, and I'm left wondering what if this will happen in a later phase of the project, or is simply not a part of this project at all. Quantitative measures are alluded to in the beginning but then never discussed again.

Response: Thank you for giving us the opportunity to clarify.

There will indeed be a quantitative component in the second phase of the project, which is a validation of the MHCP-t. This will include exploration of the distribution of the responses overall and by subgroups, testing of structural validity using principal component analysis and measurement

invariance testing or differential test functioning and measuring the tool's internal consistency. The validation strategy through will be published separately.

At this development stage, we will only explore the distribution of responses overall and by different subgroups within and across counties as well as the results on the satisfaction surveys completed by the respondents.

With respect to composite scores, we have had extensive discussions with key stakeholders about this and made an informed decision not to include any composite scoring as it is not acceptable to stakeholders within the countries Categorization of countries, or institutions within a country, can lead to unnecessary comparisons. The MHCP-t is to be utilized as a mapping and reflective exercise on health data information systems, migration-related policies in place and main aspects of migration-related healthcare provision to strengthen systems within countries, and not to name and shame.

On p.13 we have now included the updated paragraphs as follows:

"The quantitative data, generated by the tool administration and the surveys during its pilot test will be analyzed on the statistical software package STATA. We will explore the distribution of responses overall and by different subgroups within and across counties as well as the results on the satisfaction surveys completed by the respondents. Statistical differences on the respondents' tool ratings on the satisfaction survey will be explored by job profile, sex and country using ordinal logistic regression. To preserve anonymity, a study code will be given to each respondent. Quantitative data will be entered into a database containing fields for each process dimension.

The MENA MH Consortium had decided not to include any composite scoring to the MHCP-t, as it is not acceptable to stakeholders categorization of countries, or institutions within a country, which lead to unnecessary comparisons. The MHCP-t is to be utilized as a mapping and reflective exercise on health data information systems, migration-related policies in place and main aspects of migration-related healthcare provision to strengthen systems within countries, and not to "name and shame"".

- I do not see a discussion of limitations, but that may not be required for protocol papers, in 2which case please ignore this comment.

Response: Two study limitations have been added (together with three methodological Strengths) under the section "Strengths and limitations of this study" (p.3). These are also explained under the new section "Strengths and limitations" (p. 15):

"While the study plans to assess mechanisms of change triggered by the participative interactions, the pilot testing phase might not be long enough to fully evaluate the tool's long-term impact on migrant health monitoring at the national and regional levels.

The development and pilot testing of the tool will take place in three countries of North Africa, the exclusion of other countries in the Middle East may limit the tool's broader applicability across the region, as the migration and health contexts can vary significantly from country to country. Upon its

successful development in the study countries, the MHCP-t will be rolled-out in other countries of the region and beyond, after appropriate contextual validation."

Reviewer 2

Dr. Gabriela Koppenol-Gonzalez, War Child Alliance
Comments to the Author:

Dear authors,

First of all, I enjoyed reviewing this manuscript very much, I think it describes very important work addressing a serious gap in global mental health and the participatory approach is very much needed. Overall, the manuscript is well written and the protocol is clear. However, while reading the manuscript some questions came up and addressing them in the text may clarify some aspects to the reader.

I suggest to expand the Introduction with a bit more explanation about the tool. The problem is clear, but how the tool will address that problem can use some additional information. For example, it is stated on pp.4-5 that the tool will focus on multiple diseases and "enable ministries of health to respond to the health needs of diverse mobile populations as they arise in real-time". How is that envisioned? Will the tool enable a link between health data and populations as they change locations? If so, how? Is the tool like a database that can be consulted to obtain this information? And how will that be done without disaggregated information on migrant population groups?

Response*:* Thank you for underlying this need of clarification in the text. We have now provided some more detail in the highlighted sentence (p.5): "with the view to enable ministries of health to respond to the health needs of diverse mobile populations as they arise in real-time by identifying gaps in health data collection, migration-related policies and healthcare provision."

Further, we added a new sentence at the end of the MHCP-t description in the Introduction (p.5):

"In practical terms, MHCP-t will operate as an iterative online survey with multiple questions that collects information on migrant indicators related to the three domains, health data, policies specifically related to migrant populations, and access to healthcare provision (example of a question related to the health data domain would be: (a) Do you have any type of migrant indicator in your dataset? (b) If yes, how are migration variables captured?). The respondents of the survey will be different type of stakeholders (e.g., NGOs, public health authorities, disease specific programmes) at country level. The survey will be eventually developed on a secure web platform, the Research Electronic Data Capture (REDCap), and the survey will be run periodically by selected national stakeholders to monitor progress across migration-related health indicators".

On p.8 under Procedure it says that the name of the NGO staff and healthcare professionals will be collected. Will the reports be anonymous by linking the names to an ID in a separate file? If so,

please mention this in the text.

Response: We have rephrased this part of the text for clarification (p.9). No names of NGO staff/ healthcare professionals will be asked, rather the name, type and location of institution/ organization will be registered. Prior to analysis all transcripts will be anonymized both for participants and their institutions/organization, if it applies:

"…while for NGO staff and healthcare professionals: country of origin, age, sex, job position in the organization, years of experience working with migrants as well as name, type and location of institution/organization. IDI and FGD will be audio-recorded after written informed consent is obtained from participants. Audio-recordings of the sessions will be destroyed once verbatim transcriptions are completed. All transcripts will be anonymized (for participants and their institutions, if applicable) before analysis".

On p.9 under Stage 2: At experts' level it says that the qualitative study results and the systematic review findings will be summarized and compared, but what will be compared? It seems as if the qualitative results will be used for two of the domains, while the review findings will be used for the third domain. I suggest to clarify here what the comparison entails.

Response: Thank you very much indeed for pointing out this aspect in the study procedure. We confirm that there is no comparison between the preliminary qualitative results and the systematic review findings. An analytical praxis will take place to translate those findings into indicators across the three domains, as it is indeed mentioned in the same paragraph (p.9):

"The analytical praxis used by the researchers will translate systematic review findings into indicators related to migrant health data and health policies, while qualitative research findings will inform indicators concerning the domain of healthcare provision. National and international experts will review the drafted items per domain".

We have now deleted "and compare" in the first sentence of the paragraph (Stage 2: At experts' level).

Minor: on p.9 line 34 it says Tunisia twice, should be Egypt?

Response: This is now corrected in the indicated sentence: "…Morocco, Tunisia and Egypt".

On p.10 second paragraph it is explained how the indicators from field level will be used as input for the expert level rating, that there is room for proposal of new indicators and that strong disagreements will be put at the plenary for group consensus. I was wondering what the procedure will be in case there are many strong disagreements between the indicators from stage 1 and those from stage 2? Could you please add that to this part?

Response: The stepwise methodology with fieldwork, systematic reviews and expert feedback allows the multilevel and multisectoral representation of all indicators in the drafted tool. Participation of

representatives from migrant communities in the Migrant Health Secretariats will bridge any arising conflicting issues between stage 1 and stage 2 of the study procedure. In this sense (p.11), we have added to the indicated section:

“Any new suggested indicators together with those which have created strong disagreements based on stages 1 and 2 of the study procedure, will be put at the plenary for final voting towards a group consensus within the MHS, which include the engagement of representatives of migrant communities”.

On p.11 line 5, could you please add that the data capture platform will be used to collect data on the tool? It only became clear to me later in the text.

Response: We have rephrased this sentence (p.11) for clarity as follows:

“The revised set of indicators will be used in the pilot version of the MHCP-t, which will be built on an electronic data capture platform”.

On p.13 line 17 it says "between the two countries", should that be "three countries"?

Response: This is now corrected on the sentence:

“…between the three countries…”

Also on line 18, it is briefly mentioned that inferential statistics will be performed and reported. I suggest to specify what type of inferential statistics and with what aim.

Response: Inferential statistics will apply to the satisfaction survey when comparing scores on the survey by respondents’ profile, sex, and country where possible using ordinal logistic regression to see if there are any differences. If we do find significant differences, we will interview relevant respondents to understand why we see this difference.

We have now rephrased the indicated paragraph (p.14) as below:

“The quantitative data, generated by the tool administration and the surveys during its pilot test will be analyzed on the statistical software package STATA. We will explore the distribution of responses overall and by different subgroups within and across counties as well as the results on the satisfaction surveys completed by the respondents. Statistical differences on the respondents’ tool ratings on the satisfaction survey will be explored by job profile, sex and country using ordinal logistic regression. To preserve anonymity, a study code will be given to each respondent. Quantitative data will be entered into a database containing fields for each process dimension.

The MENA MH Consortium had decided not to include any composite scoring to the MHCP-t, as it is not acceptable to stakeholders the categorization of countries, or institutions within a country, which may lead to unnecessary comparisons. The MHCP-t is to be utilized as a mapping and reflective exercise on health data information systems, migration-related policies in place and main aspects of migration-related healthcare provision to strengthen systems within countries, and not to "name and shame"".

As we clearly mention on p.12: "The final version of the tool will be ready for its statistical validation (validation strategy to be published separately)".

I'm very happy to see the part about migrants' and stakeholders' involvement on p.13 and encourage you to write about that too, after conducting the study, Best of luck!

Response: Thank you so much for this suggestion.

VERSION 2 - REVIEW

Reviewer 1

Name Espinoza, Juan

Affiliation Ann and Robert H Lurie Children's Hospital of Chicago, Stanley Manne Children's Research Institute

Date 28-Oct-2024

COI

The authors have done a nice job of responding to most of the comments.

My only concern is related to how broadly the authors intend to apply the term "migrant". In my first round of comments, I asked them clarify the definition of migrant, and why it makes sense to look at them all these different communities together. the authors responded (to paraphrase) that the IOM uses a broad definition, and therefore they will use a broad definition. To me, this makes the tool less useful and less aligned with the stated goals of the authors. The authors explicitly state that tool is intended to address major health disparities in the region, and that the tool will "specifically have a transversal focus on multiple diseases (tuberculosis (TB), HIV, Hepatitis B/C, Neglected Tropical Diseases (NTD), malaria, maternal and neonatal health (MHNH), non-communicable diseases (NCD), mental health), vaccine-preventable diseases and vaccination coverage."

I agree and wholeheartedly endorse this focus, but the use of the terms "migrant" as broadly as defined by the IOM, means that it includes, for example, the 80,000 relatively wealthy Europeans that live in Casablanca, Rabat, and Marrakech. These individuals do not face

major health inequities (in fact they have access to some of the best healthcare in Morocco), and are not particularly impacted by TB, HIV, NTD, etc.

My point is that the authors either need refine their definition of migrants to exclude wealthy expat communities to be internally consistent with their stated aims, or include them and then explain why it is valuable to include them in a tool that essentially is not applicable to them. This will not be a problem unique to Morocco; there are large, wealthy expat communities in UAE, Saudi Arabia, Bahrain, Egypt, and Jordan.

**Reviewer**	**2**
**Name**	**Koppenol-Gonzalez, Gabriela**
**Affiliation**	**War Child Alliance , Research & Development**
**Date**	**11-Nov-2024**
**COI**	

Dear authors,

Thank you for addressing my previous comments, all the responses are clear.

Best wishes

VERSION 2 - AUTHOR RESPONSE

Reviewer: 1
Dr. Juan Espinoza, Ann and Robert H Lurie Children's Hospital of Chicago

Comments to the Author:

The authors have done a nice job of responding to most of the comments.

My only concern is related to how broadly the authors intend to apply the term "migrant". In my first round of comments, I asked them clarify the definition of migrant, and why it makes sense to look at them all these different communities together. the authors responded (to paraphrase) that the IOM uses a broad definition, and therefore they will use a broad definition. To me, this makes the tool less useful and less aligned with the stated goals of the authors. The authors explicitly state that tool is intended to address major health disparities in the region, and that the tool will "specifically have a transversal focus on multiple diseases (tuberculosis (TB), HIV, Hepatitis B/C, Neglected Tropical Diseases (NTD), malaria, maternal and neonatal health (MHNH), non-communicable diseases (NCD), mental health), vaccine-preventable diseases and vaccination coverage."

I agree and wholeheartedly endorse this focus, but the use of the terms "migrant" as broadly as defined by the IOM, means that it includes, for example, the 80,000 relatively wealthy Europeans that live in Casablanca, Rabat, and Marrakech. These individuals do not face major health inequities

(in fact they have access to some of the best healthcare in Morocco), and are not particularly impacted by TB, HIV, NTD, etc.

My point is that the authors either need refine their definition of migrants to exclude wealthy expat communities to be internally consistent with their stated aims, or include them and then explain why it is valuable to include them in a tool that essentially is not applicable to them. This will not be a problem unique to Morocco; there are large, wealthy expat communities in UAE, Saudi Arabia, Bahrain, Egypt, and Jordan.

Response:

Thank you very much for underlying this very important issue.

Under the section of “Strengths and Weaknesses” (p.15-16) we have now included the following:

“We do recognize that the migrant definition we use is very broad and it may include migrant groups that are not of primary interest. However, our tool is not intended to address the health burden of vulnerable migrants, but rather to map how the indicators needed to do this assessment are being collected throughout each country. Therefore, we used the broad IOM migrant definition, since “being a migrant” may be easier to be captured in health information systems and/or health surveillance systems. In other words, it is not easy to differentiate expatriates vs other groups of migrants unless the country of birth or last country of residence is collected, since health information systems usually do not reflect other vulnerability indicators per se (e.g. poverty).

Our tool precisely aims to explore any type of migrant indicators, and further, how such indicators are collected and whether they are able to capture vulnerabilities among different migration groups. For example, the indicator “country of birth” would be able to differentiate people from high vs low-income countries, and “migrant status” would assess the information related to the legal status of the migrants. The purpose of the tool is that in the long-term migrant indicators of surveillance systems and datasets can be redefined by selecting those appropriate to capture adequately the migrant vulnerabilities across different migrant groups.”.

VERSION 3 - REVIEW

**Reviewer**	**1**
**Name**	**Espinoza, Juan**
**Affiliation**	**Ann and Robert H Lurie Children's Hospital of Chicago, Stanley Manne Children’s Research Institute**
**Date**	**02-Jan-2025**

COI

Thank you for adding the new section discussing the limitations of the definition of migrants. This addresses my comments appropriately. I have no further edits.

**Reviewer**	**2**
**Name**	**Koppenol-Gonzalez, Gabriela**
**Affiliation**	**War Child Alliance , Research & Development**
**Date**	**09-Dec-2024**
**COI**	

Dear authors,

Thank you for the clarifications and adjustments in the paper.

Kind regards.
